# Reduced vagal tone in women with endometriosis and auricular vagus nerve stimulation as a potential therapeutic approach

**DOI:** 10.1038/s41598-020-79750-9

**Published:** 2021-01-14

**Authors:** Meihua Hao, Xishi Liu, Peijing Rong, Shaoyuan Li, Sun-Wei Guo

**Affiliations:** 1 Shanghai Obstetrics and Gynecology Hospital, Fudan University, Shanghai, 200011 China; 2 Shanghai Key Laboratory of Female Reproductive Endocrine-Related Diseases, Fudan University, Shanghai, China; 3 Institute of Acupuncture and Moxibustion, China Academy of Chinese Medical Sciences, Beijing, 100700 China

**Keywords:** Diseases, Reproductive disorders, Endocrine reproductive disorders, Translational research

## Abstract

Sensory and sympathetic nerves have been shown to promote the progression of endometriosis through the release of neuromediators and the lesional activation of respective receptors. The role of vagus nerves (VN) in lesional progression, however, is completely unclear, despite the signs suggestive of increased sympathetic tone in women with endometriosis. This study was undertaken to investigate whether VN plays any role in the progression of endometriosis. We recruited 45 patients with endometriosis and 42 healthy women, who were given electrocardiogram test and their heart rate variability was evaluated. In addition, three prospective, and randomized mouse experiments were conducted that evaluated, respectively, the effect of vagotomy, the effect of VN stimulation (VNS), and the therapeutic potential of VNS after the endometriosis was well established. All lesions were excised, weighed, and processed for immunohistochemistry and histochemistry analysis of select markers for lesional progression and fibrosis. We found that endometriosis patients exhibited reduced vagal activity as compared with controls, indicative of disrupted autonomic balance. Vagotomy increased while VNS decreased the lesion weight as compared with control mice, concomitant with more progressive and retarded lesion development and fibrogenesis, respectively. In addition, VNS demonstrated promising therapeutic effect, as evidenced by significantly reduced lesion weight, more attenuated lesional progression concomitant with improved hyperalgesia. Taken together, our data indicate that VN activity may play a dampening role in the progression of endometriosis. Consequently, boosting the VN activity may have therapeutic potentials for patients with endometriosis.

## Introduction

Endometriosis, characterized by the deposition and growth of endometrial-like tissues outside of uterine cavity, is a benign and a debilitating gynecological disease affecting 6–10% of women of reproductive age^1^. It is an estrogen-dependent disease, and also features with chronic inflammation^1^. Despite years of research, our understanding of its etiology, pathogenesis and pathophysiology is still fragmentary. As a result, its effective treatment still remains a challenge^2^, and the development of novel non-hormonal therapeutics has been painfully stagnant^3^.

In the last few years, evidence has been accumulating that endometriotic lesions are wounds undergoing repeated tissue injury and repair (ReTIAR) owing to cyclic bleeding of ectopic endometrium^4–6^. As wounds, endometriotic lesions are highly vascularized (at least initially)^7^ and richly innervated^8,9^. However, it is well known that neuromediators secreted by sensory and autonomic nerves are implicated in all phases of tissue repair^10^. For example, substance P (SP) released by sensory nerves on the wounding site engenders vasodilatation and vascular permeability promoting plasma extravasation^11,12^, mediated by nitric oxide and histamine induced by SP via its receptor, neurokinin receptor 1 (NK1R), present on both endothelial cells and mast cells^13,14^. Sensory nerve derived calcitonin gene related-protein (CGRP) also is implicated in vasodilatation and inflammation^15^. Both SP and CGRP can modulate collagen production and matrix metallopeptidase 2 (MMP-2) and MMP-9 activities during skin wound healing^16^. SP accelerates the normal acute and chronic wound healing processes^17–19^, while sensory denervation impairs cutaneous wound healing through increased apoptosis, and reduced proliferation and wound contraction^20–22^. Similarly, sympathetic denervation by oxidopamine also results in impaired wound healing which was associated with a decrease of neurogenic inflammation^23,24^.

Nerves are a notable feature of the lesional microenvironment, especially in deep endometriosis^25–28^. In uncanny similarity to wound healing, we have previously reported that sensory nerve derived SP and CGRP can accelerate the progression of endometriosis^29,30^, so can β2 adrenergic receptor (ADRB2) agonists^31^ and NK1R agonist^29^. These results clearly demonstrate that the promotional roles of both sensory and sympathetic nerves in the development of endometriosis.

However, the visceral nerves innervating the abdominal/pelvic cavity are not confined exclusively to sensory and sympathetic nerves. Within several nerve plexuses in the pelvic cavity, there are also parasympathetic nerves, which consist of mostly cholinergic vagus nerves (VN)^32^. The activities of the sympathetic and VN are in dynamic balance in normal physiological conditions, and their complex interplay is of critical importance in maintaining the homeostasis of vital functions such as breathing and heartbeat. For the cardiovascular system, a cranked up sympathetic tone, i.e. an autonomic imbalance characterized by domination of the sympathetic branch of autonomic nervous system over parasympathetic branch^33^, has been identified as characteristic of several cardiovascular diseases while activated VN is viewed as beneficial^34^. There is accumulating evidence that suggests the autonomic imbalance featuring a hyperactive sympathetic system and a depressed VN system is associated with a plethora of pathological conditions such as hypertension^35^ and heart failure^36^, and is one of the most powerful predictors of death^37^.

From a broader perspective, the importance of the VN system actually goes well beyond the dynamic autonomic balance. In the last two decades, it has been recognized that there is a complex and intricate crosstalk between the nervous and immune systems through a complex set of cytokines, neurotransmitters and hormones, which serves as counter-regulatory mechanisms capable of dampening inflammation and restoration of homeostasis^38,39^. In particular, following the discovery that VN stimulation (VNS) effectively attenuates the systemic inflammatory response to endotoxin^40^, the concept of cholinergic anti-inflammatory pathway (CAIP) was subsequently proposed^41^. The CAIP proposition postulates that there is an intricate link between the nervous and immune systems. Inflammatory mediators resulting from either pathogen invasion or tissue injury activate sensory nerves and transduce signals concerning the state of inflammation to the central nervous system, which release, through efferent nerves, neuromediators that act on immune cells and modulate local inflammation to restore local immune homeostasis^42^. Within the CAIP, the VNS plays a pivotal role since its activation, followed by the activation of the α7 nicotinic acetylcholine receptor (α7nAChR), potently reduces inflammation in peripheral tissues^43,44^.

Given that the VN densely innervate various visceral organs within the abdominal cavity where most endometriotic lesions are located, it is perhaps timely to investigate the role of VN in lesional progression, which so far has been completely unknown. Since lesions are fundamentally wounds undergoing ReTIAR, deciphering cellular and molecular mechanisms involved in this ReTIAR is of paramount importance for the effective treatment of endometriosis. In particular, understanding whether VN has any role in the ReTIAR should help us understand why there is a persistent and chronic inflammation in endometriosis. In this study, we first investigated whether there is a sympathetic-VN imbalance in women with endometriosis. Then, we employed a mouse model of endometriosis to see whether suppression or induction of VN activity has any effect on lesional progression.

### Results

#### The sympatho-vagal imbalance in women with endometriosis

We first investigated the state of sympatho-vagal balance through the evaluation of heart rate variability (HRV) in patients with surgically and histologically diagnosed ovarian endometrioma (OE) and in age-matched normal volunteers. HRV has been used extensively as an indicator of imbalances within the autonomous nerve system. The characteristics of the recruited patients with OE and healthy women are listed in Table 1.

**Table 1:** Characteristics of recruited subjects in this study.

Variable	Healthy subjects (n = 42)	Patients with ovarian endometriomas (n = 45)	*p* value
**Age (in years)**			
Mean ± S.D	33.6 ± 7.6	33.0 ± 6.6	0.99
Median (range)	32(23–49)	32(22–48)	
**Menstrual phase**			
Proliferative	ND	22 (48.9%)	NA
Secretory		23 (51.1%)	
**Parity**			
0	20 (47.6%)	23 (51.1%)	0.71
1	18 (42.9%)	20 (44.4%)	
≥ 2	4 (9.5%)	2 (4.4%)	
**rASRM score**			
Mean ± S.D	NA	38.8 ± 16.97	NA
Median (range)		48(4–76)	
**rASRM stage**			
I	NA	1 (2.2%)	NA
II		0 (0.0%)	
III		24 (53.3%)	
IV		20 (44.4%)	
**Severity of dysmenorrhea**			
None	35(83.3%)	19 (43.2%)	0.0003
Mild	4 (9.5%)	19 (43.2%)	
Moderate	3 (7.1%)	6 (13.3%)	
Severe	0 (0.0%)	1 (2.2%)	
**Co-occurrence with uterine fibroids**			
No	36 (85.7%)	34 (75.6%)	0.29
Yes	6 (14.3%)	11 (24.4%)	
**Co-occurrence with adenomyosis**			
No	42 (100.0%)	39 (86.7%)	0.056
Yes	0 (0.0%)	6 (13.3%)	
**Co-occurrence with deep endometriosis**			
No	42 (100.0%)	43 (95.6%)	0.49
Yes	0 (0.0%)	2 (4.4%)	

*rASRM* revised American Society for Reproductive Medicine classification system for endometriosis, *ND* not determined, *NA* not applicable.

HRV can be analyzed in both time and frequency domains. In particular, vagal and sympathetic activities are analyzed by HRV can be characterized by several parameters, such as the root mean square of successive differences (RMSSD), percentage of differences between adjacent R-R intervals > 50 ms (pNN50 (%)), normalized high-frequency (HF) power, normalized low-frequency (LF) power and frequency power ratio LF/HF. RMSSD, pNN50 and HF all reflect the vagal tone, but RMSSD typically provides a better assessment and it is often preferred to pNN50^45^. In contrast, LF reflects a mix between sympathetic and vagal influences that shows an influence of both sympathetic and parasympathetic branches, and the LF/HF ratio also has been considered as indicative of sympathetic to parasympathetic autonomic balance^45^.

**Figure 1:**
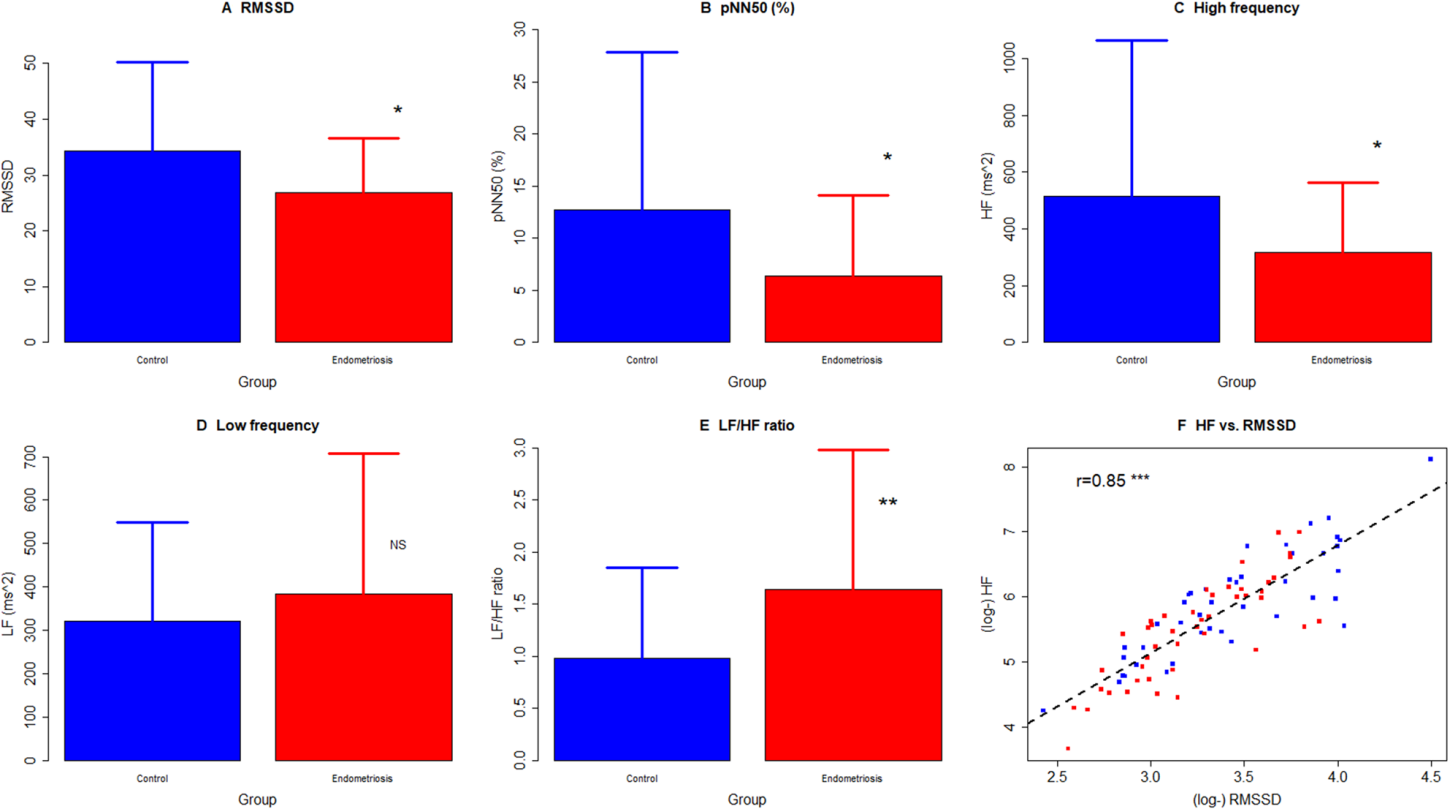
Summary of heart rate variability (HRV) measurements between patients with ovarian endometriomas (OE) and healthy women (Controls). Total RMSSD (**A**), pNN50 (%) (**B**) and HF (**C**) are reduced significantly in patients with endometriosis as compared with controls. (**D**) The difference in LF did not reach statistical significance. (**E**) The mean LF/HF ratio is significantly higher in patients with endometriosis patient as compared with controls. (**F**) Scatter plot showing the relationship between HF and RMSSD, both log-transformed. Each red and blue dot represents one data point from patient with endometriosis and controls, respectively. The dashed line represents the regression line. The Pearson’s correlation coefficient is shown, along with its statistical significance level. Symbols for statistical significance levels: NS: *p* > 0.05; *p < 0.05; **p < 0.01; ***p < 0.001.

Compared with normal women, there was a significant reduction in RMSSD, pNN50 (%) and HF in women with OE (all 3 *p*’s < 0.035; Fig. 1A-C). Multiple linear regression incorporating age, parity, presence or absence of fibroids, severity of dysmenorrhea and having or not endometriosis confirmed these findings (*p*’s < 0.012 in all three regression analyses). While women with OE appeared to have higher LF than controls, the difference did not reach statistical significance (*p* = 0.43; Fig. 1D). However, the LF/HF ratio was significantly elevated in women with endometriosis as compared with controls (*p* = 0.0067 by Wilcoxon’s test, or *p* = 0.0031 by linear regression; Fig. 1E).

As expected^46^, RMSSD correlated closely with HF (r = 0.85, *p* < 2.2 × 10^−16^; Fig. 1F). In addition, pNN50 correlated positively with both RMSSD (r = 0.85, *p* < 2.2 × 10^−16^) and HF (r = 0.81, *p* < 2.2 × 10^−16^), **as previously reported^47^.

Within women with OE, none of the above measurements was found to be associated with the rASRM scores, menstrual phase, or severity of dysmenorrhea (all *p*’s > 0.10). However, advanced age appeared to be associated with reduced pNN50, HF and LF (all *p* values < 0.035). Since RMSSD, pNN50, and HF all provide assessment of vagal tone (especially RMSSD), while LF and the LF/HF ratio measure the mix of sympathetic and vagal activity^45^. Our data, taken together, indicate that patients with OE exhibited decreased vagal activity yet increased sympathetic activity, indicating the sympatho-vagal balance was disrupted with reduced vagal tone.

#### Vagotomy accelerates the development of endometriosis in mice

Given the finding of reduced vagal activity in women with endometriosis, one immediate question would be: is it the cause or consequence of endometriosis? To address this question, we conducted a mouse experiment to see whether vagotomy, as a way to mimic depressed vagal activity, can accelerate the progression of endometriosis. We first performed the vagotomy procedure in 10 female Balb/C mice. For control mice, we performed exactly the same procedure as the vagotomy group except the actual vagotomy. Two weeks after the vagotomy procedure, endometriosis was induced through intraperitoneal injection of uterine fragments as described previously^31^.

**Figure 2:**
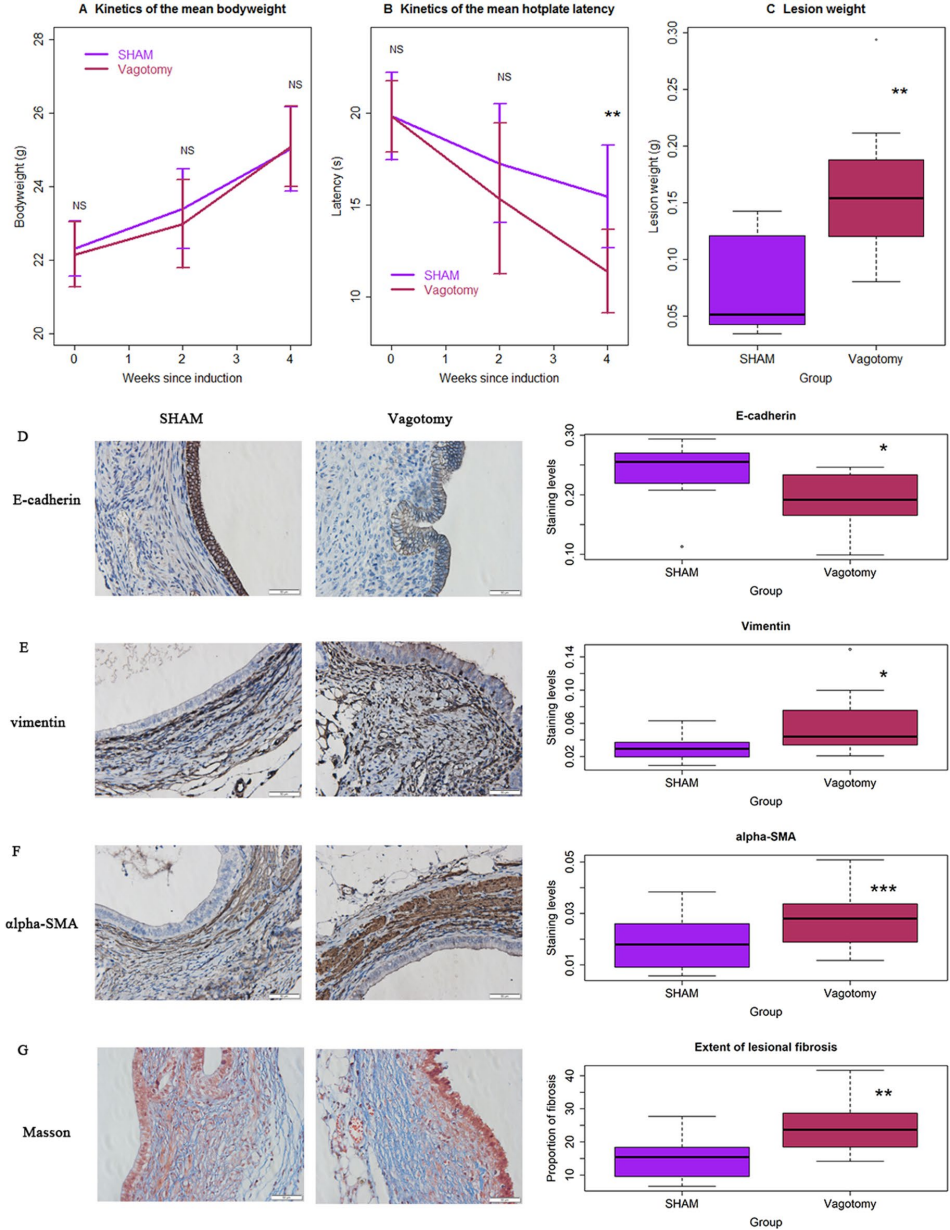
Vagotomy accelerates the development of endometriosis in mouse. (**A**) Kinetics of the mean bodyweight in the vagotomy and the control groups. No significance was found between two groups at any time point evaluated. (**B**) Kinetics of the average hotplate latency, tested at the indicated times in the two groups. Statistically significant difference in latency between the two groups is found at the end of the experiment, 2 weeks after the induction of endometriosis. (**C**) Boxplot of the lesion weight between mice with vagotomy and without. Representative immunostaining results for E-cadherin (**D**), vimentin (**E**) and α-SMA (**F**) in ectopic lesions from the two groups of mice (left panel), along the boxplot summarizing the immunostaining data (right panel). (**G**) Representative images of Masson trichrome staining in endometriotic lesions in vagotomy and control groups (left panel), along with the boxplot summarizing the results (right panel). The smooth muscle cells are stained in red and collagen fibers in blue. n = 10 in each group. The asterisk indicates the statistical significance level, calculated based on multiple linear regression analyses: NS: *p* > 0.05; *p < 0.05; **p < 0.01; ***p < 0.001. In all regressions, *R*^*2*^≧0.22.

No mouse died during the entire experiment period. No difference in bodyweight was found between the two groups before, or 2 and 4 weeks after the induction (all 3 *p* values > 0.46; Fig. 2A). As expected, there was no significant difference in hotplate latency prior to the vagotomy procedure (*p* = 1.0, Fig. 2B). While the latency in mice with vagotomy appeared to be shorter than the control mice 2 weeks after the procedure, the difference did not reach statistical significance (*p* = 0.28; Fig. 2B). The latency in the former, but not latter, group was significantly reduced as compared with the baseline levels (*p* = 0.027 vs. *p* = 0.13). The induction of endometriosis further reduced the latency in both groups (both *p*’s < 0.016), but mice with vagotomy had significantly shorter latency than those without (*p* = 0.007; Fig. 2B). Consistent with the more reduction in hotplate latency, the average lesion weight in mice with vagotomy was more than 2 folds heavier than that in mice without (163.5 ± 60.1 mg vs. 72.0 ± 42.0 mg; *p* = 0.0021; Fig. 2C), even though no difference in the number of lesions between the two groups was found (3.2 ± 1.5 vs. 2.3 ± 1.1, *p* = 0.15).

Epithelial-mesenchymal transition (EMT), fibroblast-to-myofibroblast transdifferentiation (FMT) and fibrogenesis are known to be key landmarks in endometriosis development^29,48^. To see whether vagotomy accelerates EMT, FMT and fibrogenesis, we performed IHC staining of E-cadherin, vimentin and α-SMA, and also evaluated the extent of lesional fibrosis by Masson trichrome staining in endometriotic lesions. In the vagotomy group, the E-cadherin staining levels in the epithelial component were significantly lower than the sham-operated mice (*p* = 0.038; Fig. 2D). In contrast, the staining levels of vimentin in the epithelial component were significantly elevated as compared with control mice (*p* = 0.029; Fig. 2E). Similarly, the α-SMA staining levels were also increased significantly in mice with vagotomy as compared with those without (*p* = 0.0004; Fig. 2F). Consistently, the extent of lesional fibrosis was significantly elevated in vagotomy group as compared with the control group (*p* = 0.0029; Fig. 2G). Notably, the extent of lesional fibrosis correlated with the staining levels of vimentin (r = 0.61, *p* = 0.0004), lesion weight (r = 0.47, *p* = 0.035) and the hotplate latency (r = 0.54, *p* = 0.014) by the end of the experiment. Thus, we provided the first piece of evidence that disruption of vagal activity through vagotomy accelerates lesional progression via promoting EMT and fibrogenesis, and likely through FMT.

#### VNS decelerates lesional progression in mice with induced endometriosis

Having found that depressed vagal activity through vagotomy could accelerate lesional progression, we wondered whether its opposite, i.e. VNS, could decelerate lesional progression. We thus conducted the second experiment to test this possibility. We performed the VNS procedure in mice in the VNS group but sham stimulation in the control group 1 day prior to the endometriosis induction procedure.

**Figure 3:**
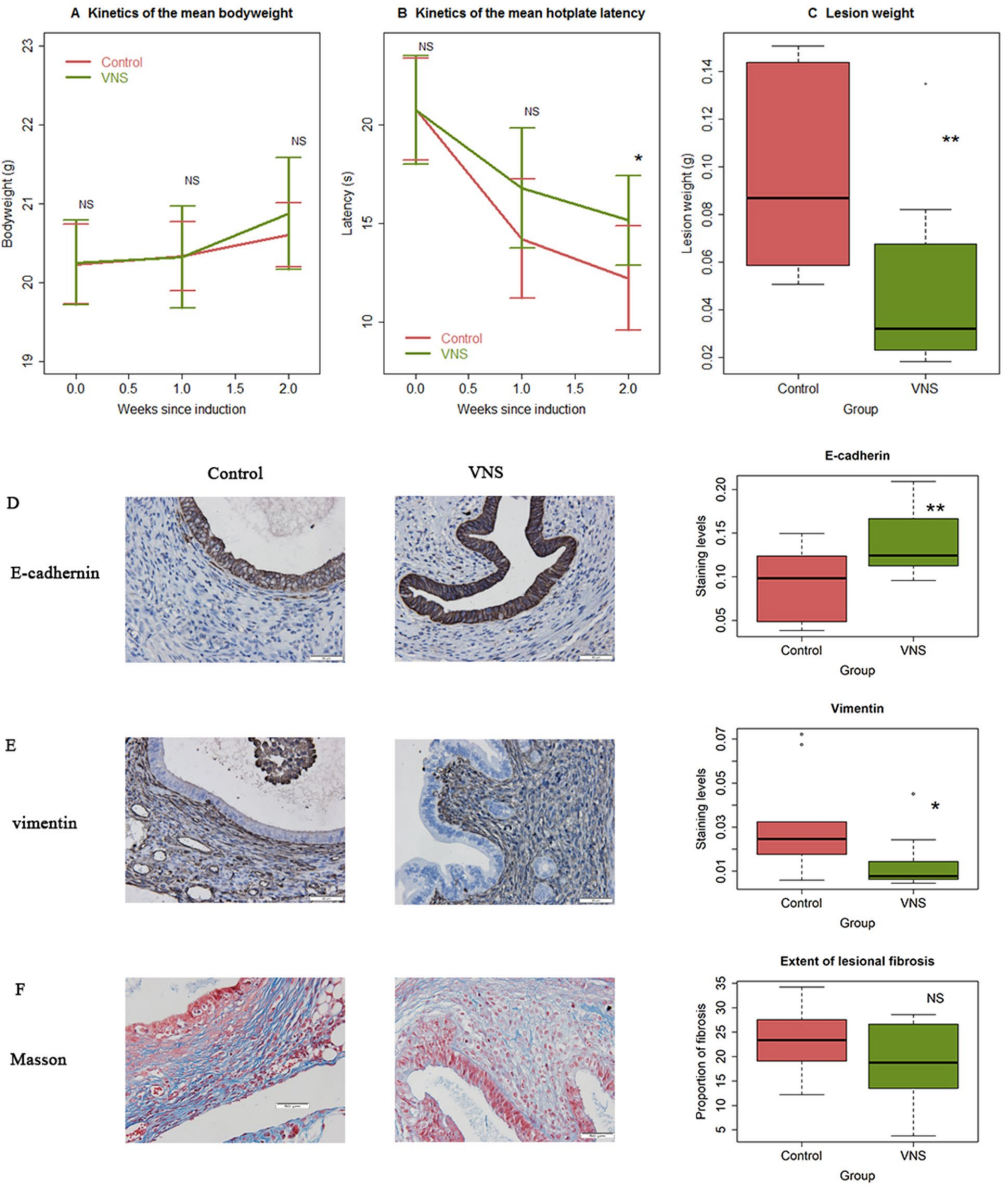
Vagal nerve stimulation (VNS) decelerates the development of endometriosis in mouse. (**A**) Kinetics of the mean bodyweight in control and VNS groups. No significance was found between two groups at any time point evaluated. (**B**) Kinetics of the mean hotplate latency, tested at the indicated times in the two groups. Statistically significant difference in latency between the two groups is found at the end of the experiment, 2 weeks after the induction of endometriosis. (**C**) Boxplot summarizing the lesion weight data between control and VNS mice. Representative immunostaining results for E-cadherin (**D**) and vimentin (**E**) in endometriotic lesions from control and VNS mice (left panel), along the boxplot summarizing the staining data (right panel). (**F**) Representative images of Masson trichrome staining in endometriotic lesions in control and VNS groups (left panel), along with the boxplot summarizing the staining data (right panel). n = 10 in each group. The asterisk indicates the statistical significance level, based on linear regression using group indicator: NS: *p* > 0.05; *p < 0.05; **p < 0.01. The *R*^*2*^ values for E-cadherin and vimentin were 0.51 and 0.27, respectively. For the exent of fibrosis, *R*^2^ = 0.10.

No difference in bodyweight was found between the two groups of mice before, and 1 and 2 weeks after the induction of endometriosis (all 3 *p*’s > 0.32; Fig. 3A). Prior to the induction, there was no significant difference in hotplate latency between two groups (*p* = 0.96; Fig. 3B). One week after the induction, mice in both groups had significantly reduced latency as a result of endometriosis (*p* = 0.002 for both groups; Fig. 3B), especially in the control group, but the difference between the two groups was not statistically significant (*p* = 0.11; Fig. 3B). However, the difference in latency became statistically significant 2 weeks after the induction (*p* = 0.029; Fig. 3B). Indeed, mice in the VNS group had their latency reduced by an average of 27.0% from the before-induction level, as compared with an average of 41.3% in the control group (Fig. 3B). Consistently, the average lesion weight in VNS mice was reduced by 50.2% as compared with the control group (47.5 ± 37.2 mg vs. 95.5 ± 40.3 mg; *p* = 0.0089; Fig. 3C), even though there was no difference in the number of lesions between the two groups (2.2 ± 0.8 vs. 2.9 ± 1.4, *p* = 0.18).

We also performed IHC staining of E-cadherin and vimentin as well as Masson trichrome staining for lesion samples of all mice. In VNS group, the E-cadherin expression levels in the glandular epithelial cells were significantly increased as compared to control group (*p* = 0.0011; Fig. 3D). In contrast, the staining levels of vimentin in the same epithelial component in VNS mice were significantly decreased as compared with control mice (*p* = 0.020; Fig. 3E). While the extent of lesional fibrosis appeared to be decreased in VNS mice as compared to control mice, the difference did not reach statistical significance (*p* = 0.19; Fig. 3F), probably due to the insufficient time for fibrogenesis to develop fully and the lack of sufficient statistical power. Thus, we provided the first piece of evidence that VNS can potentially suppress the progression of endometriosis.

#### VNS has therapeutic potential in mice with well-established endometriosis

Given the evidence that VNS, instituted shortly before the induction of endometriosis, decelerated the lesional progression, we next hypothesized that VNS also has therapeutic potential for established endometriosis. To test this hypothesis, we instituted VNS two weeks after the induction of endometriosis—when lesions were well-established- and the treatment lasted for two weeks. As controls, the mice received sham stimulation and were thus untreated.

**Figure 4:**
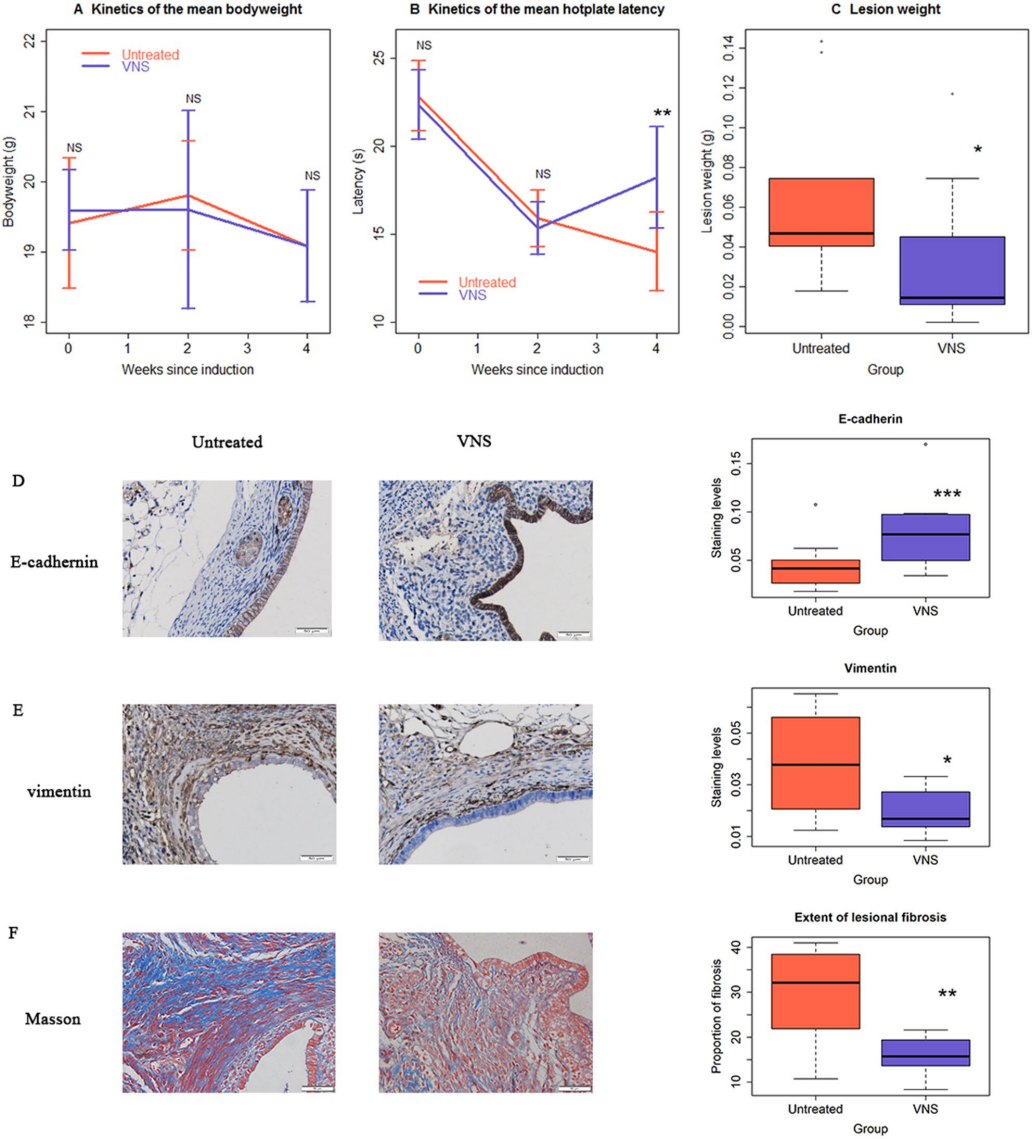
Vagal nerve stimulation (VNS) demonstrates its therapeutic potentials in mouse with well-established endometriosis. (**A**) Kinetics of the mean bodyweight in VNS and the untreated groups. No significance was found between the two groups at any time point evaluated. (**B**) Kinetics of the mean hotplate latency, tested at the indicated time points, in the two groups. Statistically significant difference in latency between the two groups is found 2 weeks after the treatment with VNS. (**C**) Boxplot summarizing the lesion weight in the control and VNS groups. Representative immunostaining results for E-cadherin (**D**) and vimentin (**E**) in endometriotic lesions from untreated and VNS-treated mice (left panel), along the boxplot summarizing the staining data (right panel). (**F**) Representative images of Masson trichrome staining in endometriotic lesions in the two groups (left panel), along with the boxplot summarizing the staining data (right panel). The smooth muscle cells are stained in red and collagen fibers in blue. n = 10 in each group. The asterisk indicates the statistical significance level, which was calculated based on multiple linear regression analyses: NS: *p* > 0.05; *p < 0.05; **p < 0.01; ***p < 0.001. In all regressions, *R*^*2*^≧0.27.

No difference in bodyweight was found between the VNS and the untreated groups before, 2 and 4 weeks after the induction (all *p*’s > 0.57; Fig. 4A). As expected, there was no significant difference in hotplate latency prior to and 2 weeks after the induction of endometriosis between two groups of mice (both *p*’s > 0.48), and both groups had significantly reduced latency 2 weeks after the induction (both *p*’s < 0.006; Fig. 4B). However, after 2 weeks of VNS treatment, mice receiving VNS had significantly longer latency than that of untreated mice (*p* = 0.0039; Fig. 4B). On average, mice receiving VNS only had their latency reduced by 18.6% since the induction of endometriosis, as compared with 38.8% in untreated mice. Compared with the latency before the treatment, the VNS mice gained 18.7% longer latency vs. further reduction by 11.9% in the untreated mice (Fig. 4B). The 2-week long treatment with VNS also resulted in an average of 50.6% reduction in lesion weight as compared with the control mice (*p* = 0.023; Fig. 4C), but the reduction in the number of lesions did not reach statistical significance (3.2 ± 1.6 vs. 3.8 ± 1.5, *p* = 0.38).

We also performed IHC analysis of E-cadherin and vimentin in the epithelial component of all lesion samples (Fig. 4D,E). We found that, compared with the untreated mice, the lesional staining of E-cadherin was significantly higher in mice with VNS treatment (*p* = 0.019; Fig. 4D). In contrast, the vimentin staining levels were significantly reduced (*p* = 0.011; Fig. 4E). Consistently, the extent of lesional fibrosis also was significantly reduced in mice receiving the VNS treatment (*p* = 0.0014; Fig. 4F). The extent of lesional fibrosis correlated positively with lesion weight (r = 0.66, *p* = 0.0016) but negatively with the hotplate latency (r =  − 0.56, *p* = 0.013). Thus, we have provided the first piece of experimental evidence that VNS treatment has therapeutic effects in mice with well-established endometriosis.

## Discussion

In this study, we have demonstrated that women with OE exhibit autonomic imbalances, featuring domination of the sympathetic branch of autonomic nervous system (ANS) over the parasympathetic branch, as evidenced by reduced vagal tone as compared with healthy women. We also have shown that vagotomy, which mimics reduced vagal activity, accelerates the progression of endometriosis in mouse. In addition, vagal stimulation can substantially decelerate lesional progression in mouse. Moreover, for mouse with well-established endometriosis, treatment with VNS significantly reduces lesion weight but not lesion number, improves endometriosis-associated hyperalgesia, and significantly restrains lesional progression and fibrogenesis.

Endometriosis is known to be a chronic inflammatory condition, featuring increased lesional infiltration of immune cells and increased production of proinflammatory cytokines and chemokines^49^. While anti-inflammation may seem to be a promising therapeutic approach^50^, clinical trials testing on the anti-inflammation therapeutics so far resulted in disappointing results^51^. This seems to suggest that focusing on the inflammation alone may not work well.

Aside from inflammation, women with endometriosis are well-recognized to show increased levels of depression^52,53^. In fact, experimental studies demonstrate that mouse with induced endometriosis exhibits increased depression^54^. It turns out that depression, in and by itself, can activate the sympathetic nervous system (SNS) and the HPA/sympathetic-adrenal-medulla (SAM) axes^55^, thus increasing the nerve fiber density in endometriotic lesions^56^ as well as the release of epinephrine and norepinephrine. This would activate ADRB2 in lesions, and the ADRB2/CREB/PKA signaling pathway^57^, resulting in the induction of focal adhesion kinase (FAK)^56,58^. This explains as why in many cancer patients, elevated depression levels are associated with reduced survival^59,60^. In endometriosis, increased release of catecholamines due to the activation of the HPA/SAM axes resulting from stress can also induce the ADRB2/CREB/PKA signaling pathway but suppress the expression of DRD2, accelerating the progression of endometriosis^31,61^.

Given the close link between endometriosis and depression, there is a question as which causes which^62^. While the elucidation of their relationship would take years of research, it is conceivable that the two can be mutually promotional. Indeed, endometriosis-related pain and/or infertility can easily generate stress, anxiety, and depression, and the latter can also exert their promotional effect on lesions through the activation of the HPA/SAM axes.

Yet alleviating depression through increasing the vagal tone may have added benefits when employed in treating endometriosis. Depression can increase the production of proinflammatory cytokines^63,64^ and reduce α7nAChR expression^65^. Inflammation also can facilitate the development of depression^66,67^. Patients with depression are reported to have reduced TNF-α levels after taking anti-depressant^68^. Thus, through boosting the vagal tone and thus activating the CAIP and improving endometriosis-related depression, it is likely that the inflammation can be effectively tamed. Indeed, VNS^69,70^ and α7nAChR agonists^40,71,72^ can effectively suppress inflammation. In particular, auricular VNS is non-invasive, inexpensive, simple, and without any serious side-effects, and has been used successfully in treating depression^73,74^.

Endometriosis-associated pain is known to have features similar to nociceptive pain, inflammatory pain and neuropathic pain^75^. For patients with long-term endometriosis, the feature of neuropathic pain is particularly more prominent^76^. Interestingly, neuropathic pain due to nerve injury is often associated with reduced α7nAChR expression, suggesting that α7nAChR may be a therapeutic target^77^. Indeed, nicotine, as an nAChR agonist, is found to effectively suppress neuropathic pain^78^. In addition, electroacupuncture has been shown to effectively suppress injury-induced expression of JAK2/STAT3 in the dorsal root ganglia through activation of α7nAChR, alleviating neuropathic pain^79,80^. Auricular VNS also has been shown to suppress neuropathic pain in rats^81^. Moreover, there are some evidence suggesting that acupuncture may be effective in treating endometriosis-related pain^82^, and this therapeutic effects may be mediated through vagal modulation of inflammatory responses in internal organs^83^.

**Figure 5:**
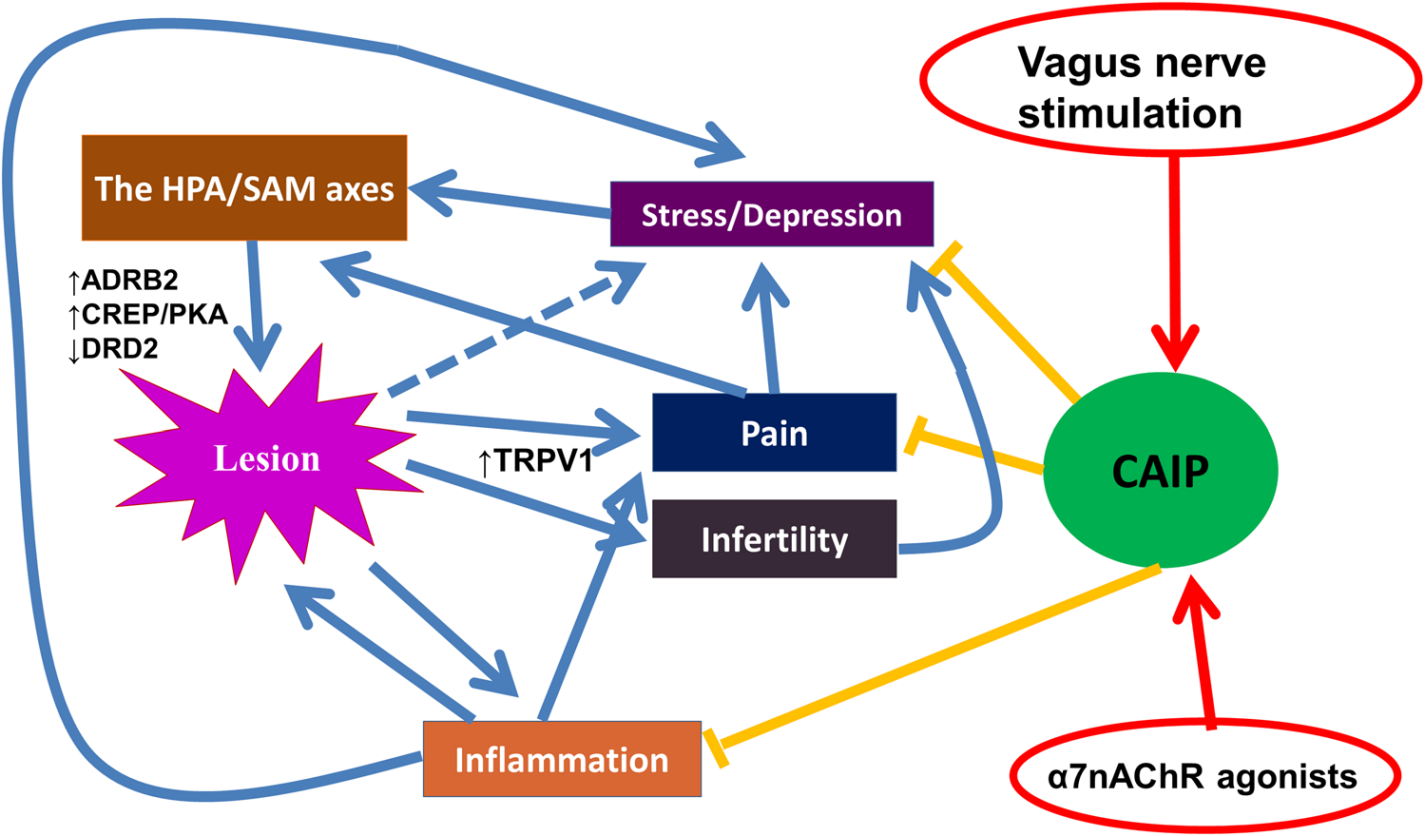
Illustrative diagram showing the possible intervention/therapeutic effects on endometriosis through the cholinergic anti-inflammatory pathway (CAIP). Endometriosis can induce pain and infertility, which can cause stress and depression, which, in turn, can lead to the activation of the HPA/SAM axes, resulting in increased release of catecholamines such as noradrenaline. The increased noradrenaline levels may induce the suppression of the DRD2 signaling pathway, the activation of the adrenergic receptor β2 (ADRB2), and the subsequent activation of the ADRB2/CREB/PKA signaling pathway, leading to increased angiogenesis and cellular proliferation, and thus lesional progression. Endometriotic lesions and inflammation are mutually stimulatory, and inflammation may also activate nociceptors such as TRPV1, facilitating pain sensation. The CAIP can be activated either by VNS or by α7nAChR agonists, leading to suppression, abrogation or attenuation of depression, pain, and inflammation, thereby achieving the therapeutic results.

Thus, compared with the stand-alone anti-inflammation through pharmacological means, VNS can simultaneously inhibit inflammation, relieve pain and improve depression. A schematic diagram showing the possible mechanisms of action for treating endometriosis by activating the CAIP is shown in Fig. 5. We note that, since both the pathophysiology of endometriosis and the molecular mechanisms underlying CAIP-mediated anti-inflammation are active research fields still with a lot of unknowns, the diagram is intended to provide a sketch of possible mechanisms with a broad stroke in order to pique the interest of our peers. It is by no means final or even complete. More research is warranted.

Our study has several strengths. First, through the combination of human study and animal experimentation, we have demonstrated that women with endometriosis have reduced vagal activity, which can promote lesional progression. Second, through experiments with vagotomy and VNS, we have shown that an important role of vagal activity in lesional progression. In addition, we demonstrated the therapeutic potential of VNS for established endometriosis.

Our study also has several limitations. First, due to the design of our study, we were unable to answer the question as whether the vagal activity can be restored after lesions are completely removed. Nor do we know whether the vagal activity in endometriosis patients is associated or affected by the severity of pain or depression levels, or the extent of lesional progression or of inflammation. Future studies are warranted to address these issues. Second, we used a mouse model of endometriosis that may not have fibrogenesis fully developed^84^. One crucial question is whether VNS is still efficacious when lesions are highly fibrotic, as in many human endometriosis. Further research is needed to address this question.

Third, we only considered OE in our study and did not include other subtypes of endometriosis such as superficial and deep endometriosis. However, accumulating evidence indicates that all endometriotic lesions are similar, in the sense that they seem to progress through EMT, FMT, smooth muscle metaplasia and fibrogenesis^85^. But one critical determinant in the tempo and pace of the progression is lesional microenvironment^5^. For example, what sets deep endometriosis apart from endometrioma is its close proximity with sensory nerves^29,30^. Understanding this helped us establish a mouse model of deep endometriosis based on a conventional endometriosis model^86^. Hence our results may be generalizable to other subtypes of endometriosis. Even if they did not, in view of the fact that OE is the most common subtype of endometriosis, our results are still meaningful.

Fourth, we selected, as controls, apparently healthy women who did not have any previous gynecological disorders and symptoms suggestive of endometriosis, or any evidence for endometriosis or adenomyosis per sonographic examination. This, of course, still cannot guarantee the exclusion of any endometriotic lesions in the pelvic cavity. This is the deficiency that almost all clinical studies of endometriosis share in common and will continue to share until there is an accurate,  reliable and non-invasive way to diagnose all subtypes of endometriosis, including microscopic or occult endometriosis. In other words, there is no easy solution for this problem in the foreseeable future. However, even if we did have this misclassification, i.e. we had unknowingly included those with asymptomatic endometriosis patients in our control group, the fact that we still found the difference in HRV between endometriosis patients and the controls group is all the more remarkable, since this would indicate that the HRV test can tolerate certain degree of misclassification and still is able to detect the difference.

Lastly, while the use of IHC provides a convenient way to ascertain the cell type, location and expression levels of select markers, this study did not provide more molecular insight into the possible mechanisms under which how VN plays a role in lesional progression. This should await more research in the future.

In summary, we have shown that women with OE have autonomic imbalances, featuring reduced vagal tone. We also have demonstrated that vagotomy accelerates endometriosis progression in mouse while VNS decelerates lesional progression in mouse. In addition, we have also shown that VNS has therapeutic potential in treating well established endometriosis in mouse. Future human studies are needed to see whether VNS can have similar therapeutic effects.

## Materials and methods

### Human subjects

This study was approved by the Ethics Review Committee of Shanghai Obstetrics and Gynecology Hospital, Fudan University, in accordance with the ethical principles spelled out in the Declaration of Helsinki and its subsequent amendments. Informed consent was obtained from all participants, and all associated methods were performed in accordance with the request of the Ethics Committee.

After informed consent, we recruited 45 patients with laparoscopicaly and histologically confirmed OE patients, who visited Shanghai OB & GYN Hospital, Fudan University, from December 2017 to December 2018, were enrolled in the study. We recruited the OE patients who were about to undergo laparoscopy due to ovarian cyst per gynecological and imaging examination. None of them had taken any anti-platelet, hormonal, oral contraceptive, or other medications at least 6 months prior to the surgery. For controls, we recruited 42 healthy women. Among them, 17 were hospital staff, and 25 were women who came to our hospital to receive the annual gynecologic check-up. None of them had any previous gynecological disorders and symptoms, or any evidence for endometriosis or adenomyosis per sonographic examination. We also excluded subjects with hypertension, heart disease, tumors, thyroid disease, and other diseases that could affect HRV. The medical records of all OE patients, including clinical symptoms and pathological reports, were carefully reviewed.

### Evaluation of HRV

To evaluate possible autonomic imbalances in women with endometriosis, we evaluated HRV, which has been used extensively for this purpose^87^. Between 8:00 and 10:00 in the morning, all recruited subjects underwent a 6-min long electrocardiogram (ECG) recording using a MicroAmbulatory ECG Recorder (DiCare-m1CP, Dimetek Digital Medical Technologies, Ltd., Shenzhen, China) following standard recommendations. The ECG Viewer software (Dimetek Digital Medical Technologies) was used to generate data PDF files and then Kubios HRV software (Kuopio, Finland) was used for HRV data analysis.

### Animals

All procedures were performed in the in-house animal facility in accordance with the guidelines of the National Research Council’s Guide for the Care and Use of Laboratory Animals^88^ and approved by the institutional review board on experimental animals at Shanghai OB/GYN Hospital, Fudan University. A total of 90 female Balb/C mice, all 6–8 weeks old and about 18–20 g in bodyweight, were purchased from Shanghai LingChang Laboratory Animal Center (Shanghai, China) and used for this study. Among these mice, 30 mice were randomly selected as donors of uterine tissues, while the remaining mice were designated as recipients.

### Induction of endometriosis

We used a well-established mouse model of endometriosis by intraperitoneal injection of uterine fragments from donor mouse^89^, as we used previously^31^.Briefly, after 7 days of acclimatization, donor mice were injected i.m. with 150 μg/kg estradiol benzoate (Animal Medicine Factory, Hangzhou, China) twice within a week. The donors were sacrificed by cervical dislocation and their uterine tissues were harvested on the day of induction. The adipose tissues, blood vessels and mesenterium surrounding the uterus were carefully removed. The uterine tissues were rinsed twice and then cut into pieces by a pair of eye scissors, with the maximum diameter of the uterine fragments being consistently smaller than 1 mm. To minimize bias, the uterine fragments made from one donor mouse were blended well in 400μLof sterile saline, divided into two equal parts, and each injected into the lower abdomen of recipient mice from two different groups using a 1 mL syringe with a 16-gauge needle. After injection, a gentle abdomen massage was given to all recipient mice to facilitate tissue dispersion and dissemination.

### The vagotomy experiment

After 2 weeks of acclimation, mice were subjected to left-sided cervical vagotomy (VX) or sham procedure adapted from van Westerloo et al.^90^. Briefly, the mouse was anesthetized by 2% chloral hydrate (w/v), and then fixed in a supine position. Its neck was shaved using a trimmer and sterilized with gauze impregnated with 75% ethanol. A 1.5-cm incision was made on the ventral cervical midline. The left carotid sheath was opened to gain exposure, and the VN was isolated and sectioned following^90,91^ (Supplementary Figure S1). The whole procedure was performed carefully and gently to avoid any damage to the carotid artery. Once VX was done the skin was closed with sutures. In sham operated mice, the left VN was exposed and isolated, but was left intact.

To see whether VX impacts on the progression of endometriosis, we first randomly divided 20 Balb/C mice into 2 equal-sized groups, the Sham and the VX. Two weeks after the procedure, endometriosis was induced by intraperitoneal injection of uterine fragments from donor mice to recipient mice, as described above. We chose 2 weeks since mice needed about 2 weeks to adapt to the change after VX^91^. The bodyweight measurement and hotplate test were administered right before the induction of endometriosis, and 2 and 4 weeks after induction. Four weeks after induction, all mice were sacrificed and all their lesions were carefully excised and harvested. The lesion weight was recorded for all mice. Immunohistochemistry analysis of E-cadherin, vimentin and α smooth muscle actin (α-SMA) was performed, with the former two evaluated within the epithelial component, and the latter, the stromal component. The extent of lesional fibrosis was evaluated by Masson’s trichrome staining.

### VNS experiments

The auricular branch of the VN extends to the pinna of the ear^92^. We used a modified Han’s accupoint nerve stimulator (HANS) (HANS-200A, Nanjing Jisheng Medical Technology Co., Ltd., Nanjing, China) to stimulate the auricular branch of the VN. HANS was connected to copper electrical wires and two electrode plates (5 mm in width). The mouse was head-fixed, with its body in a holding box in a prone position. The electrodes were fixed on the ear lobe of the auricle by two round magnets (5 mm in diameter), one each at the two sides of the ear so that the electrodes were always in close contact with the skin (Supplementary Figure S2). The reduction in heart rate was used to assess the effectiveness of VNS, where stimulation frequency was increased until an average of 10% reduction in heart rate was achieved^93^. The VNS frequency and current were thus set to 2/30 Hz at 1 mA. In the VNS group, mice received the VNS for 15 min per day, while for mice in the control group the electrodes were placed exactly in the same place, the same manner and were given same duration of manipulation just as the VNS group except without any electric current.

To see whether VNS impedes the progression of endometriotic lesions, we randomly divided 20 mice after two weeks of acclimation into 2 equal-sized groups: the control and the VNS groups. The VNS was started 1 day before the induction of endometriosis. The bodyweight and hotplate test were administered right before induction, and 1 and 2 weeks after induction. All mice were sacrificed two weeks after induction, and their lesions were carefully excised and harvested. The lesion weight was recorded. The lesional staining levels of E-cadherin and vimentin in the epithelial component were evaluated by IHC, and the extent of lesional fibrosis was quantified by Masson’s Trichrome staining.

To see whether VNS has any therapeutic potential, 20 mice were first induced with endometriosis as described above after 2 weeks of acclimation. Two weeks after the induction, the mice were randomly divided into two equal-sized groups: the control and VNS treatment group. Endometriosis was induced as described above, and VNS was carried out, with the control mice only received a sham VNS. Two weeks after the VNS treatment, all mice were sacrificed and their lesions excised and harvested. The bodyweight and hotplate test were administered right before the induction, right before VNS and two weeks after VNS. The lesional staining levels of E-cadherin and vimentin in the epithelial component were evaluated by IHC, and the extent of lesional fibrosis was quantified by Masson’s Trichrome staining.

### Immunohistochemistry (IHC)

All specimens were fixed in 4% paraformaldehyde (w/v), and then paraffin embedded. Serial 4 μm sections were fixed on the adhesive slides. Masson trichrome staining was performed as we previously described^30^. The slides were de-paraffinized in xylene and then rehydrated in alcohol of serial concentrations and used for IHC analysis for E-cadherin (1:100, Cell Signaling Technology, Boston, MA, USA), α-SMA (1:100, Abcam, Cambridge, UK) and vimentin (1:100, Abcam, Cambridge, UK). We chose to perform IHC analysis of E-cadherin and vimentin in the epithelial component of endometriotic lesions simply because both are markers of EMT^4,94^. Similarly, α-SMA is a marker for myofibroblasts and can be taken as a marker for FMT^4,94^. Markers of EMT and FMT, along with the quantification of lesional fibrosis, can be used to gauge the development stages of endometriotic lesions^6,94^.

Slides were immersed in citrate buffer and heated at 100 °C pressure cooker for 30 min, then cooled down to room temperature. The sections were incubated with the primary antibodies overnight at 4 ℃ and incubated with the secondary antibodies for 30 min at room temperature the following day. Positive staining was visualized using 3, 3′-diaminobenzidine (JieHao Biological Technology, Shanghai, China) and counterstained with hematoxylin (JieHao). For positive controls, mouse kidney tissues were used for vimentin, human breast cancer tissues for E-cadherin, and uterus tissues of adenomyosis mouse for α-SMA. For negative controls, rat serum (Boster, Wuhan, China) was used instead of primary antibodies.

The representative IHC results for positive and negative controls are shown in Supplementary Figure S3. Five randomly selected images at 400 × magnification of each sample were taken and the mean density was acquired by Image Pro-Plus 6.0 (version 6.0.0.206, Media Cybernetics, Inc, Bethesda, MD, USA).

The human breast cancer tissues were obtained from the archived tissue collection, Dept. of Pathology, Shanghai Obstetrics and Gynecology Hospital, Fudan University, and used as positive controls for E-cadherin in this study, which was approved by the Ethics Review Committee of Shanghai Obstetrics and Gynecology Hospital. No specific tissue was taken deviating from the routine therapy and no experiments were performed on patients with breast cancer. Since all tissue samples were de-identified without any personal information, the Ethics Review Committee stated that no ethical concerns are raised by the methods applied and approved this study.

### Hotplate test

Hotplate test was performed with Intelligent Hot Plate Instrument (RB-200, Chengdu Techman Software Co. Ltd., Chengdu, China) to assess the extent of endometriosis-associated hyperalgesia. The metal plate was 190 mm × 190 mm in size and the surface of the plate was heated and then kept to the temperature of to 55 ± 0.11 °C. Before the test began, mice were brought to the testing room and allowed to acclimatize for 10 min. The withdrawal latencies were determined from the moment mice were placed in the center of the metal plate. The criteria of withdrawal were licking its hind paws, quickly agitating the hind paws or jumping off the hot plate. The latency was calculated by averaging two readings recorded separated in a 24-h interval.

### Statistical analysis

The comparison of continuous variables between two groups was evaluated by Wilcoxon’s test. The paired Wilcoxon’s test was used when the before–after (induction of endometriosis) comparison was made for the same group of subjects. Pearson’s correlation coefficient was used when evaluating correlations between 2 continuous variables. Multiple linear regression was used to evaluate possible association with various HRV parameters for age, parity, severity of dysmenorrhea, and, within patients with OE, rASRM scores, presence or absence of adenomyosis/deep endometriosis, and menstrual phase. It was also used to evaluate whether the immunostaining levels and the extent of lesional fibrosis were associated with experimental procedures, along with lesion weight and number. *p* values of less than 0.05 were considered statistically significant. All computations were made with R 4.0.2 (29) (www.r-project.org).

## Supplementary Information

Supplementary Information 1.

## Abbreviations

α7nAChR: α7 nicotinic acetylcholine receptor
ADRB2: Adrenergic receptor β2
CAIP: Cholinergic anti-inflammatory pathway
CREB: cAMP responsive element-binding protein
DRD2: Dopamine D2 receptor
HPA: Hypothalamic–pituitary–adrenal
PKA: Protein kinase A
SAM: Sympathetic-adrenal-medulla
TRPV1: Transient receptor potential vanilloid type 1
