# Supplementary material for: Reduced vagal tone in women with endometriosis and auricular vagus nerve stimulation as a potential therapeutic approach
Source: Sci Rep. 2021 Jan 14;11:1345. doi: 10.1038/s41598-020-79750-9 (PMC7809474; doi:10.1038/s41598-020-79750-9)
Supplement: Supplementary file 1 — Supplementary Information 1. [file 41598_2020_79750_MOESM1_ESM.docx]

**Reduced Vagal Tone in Women with Endometriosis and**

**Aricular Vagus Nerve Stimulation as a Potential Therapeutic Approach**

**Meihua Hao, Xishi Liu, Peijing Rong, Shaoyuan Li, and Sun-Wei Guo**

**Supplementary Figures**

**
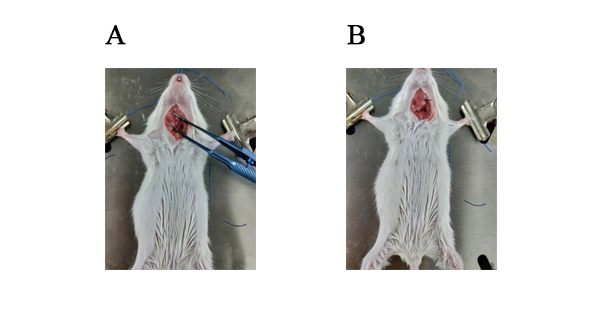
**

**Figure S1.** Representative images showing the left side cervical vagotomy. (A) A 1.5-cm incision was made on the ventral cervical midline. The left carotid sheath was opened up to gain exposure, and the vagus nerve was isolated. (B) The vagus nerve was sectioned.

**
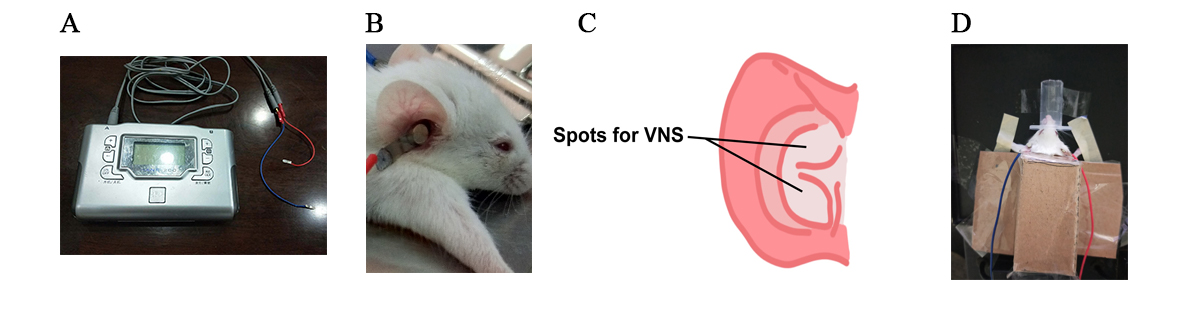
**

**Figure S2.** The instrument used for vagus nerve stimulation (VNS) procedure. (A) The commercially available modified Han’s acupoint nerve stimulator (HANS). (B) Electrodes were secured on the mouse’s ear lobe, in the auricular vagus nerve region, by two round magnets (5 mm in diameter), one each on each side of the lobe. (C) The auricular branch of the vagus nerve extends to the pinna of the ear. (D) The mouse was head-fixed, with its body in a holding box in prone position, and then was given the VNS. Panel C was adapted from our previous paper, [Shuxing Wang](https://pubmed.ncbi.nlm.nih.gov/?sort=date&term=Wang+S&cauthor_id=25880500), [Xu Zhai](https://pubmed.ncbi.nlm.nih.gov/?sort=date&term=Zhai+X&cauthor_id=25880500), [Shaoyuan Li](https://pubmed.ncbi.nlm.nih.gov/?sort=date&term=Li+S&cauthor_id=25880500) , [Michael F McCabe](https://pubmed.ncbi.nlm.nih.gov/?sort=date&term=McCabe+MF&cauthor_id=25880500), [Xing Wang](https://pubmed.ncbi.nlm.nih.gov/?sort=date&term=Wang+X&cauthor_id=25880500), [Peijing Rong](https://pubmed.ncbi.nlm.nih.gov/?sort=date&term=Rong+P&cauthor_id=25880500). Transcutaneous vagus nerve stimulation induces tidal melatonin secretion and has an antidiabetic effect in Zucker fatty rats. *PLoS One*. 2015 Apr 16;10(4):e0124195. doi: 10.1371/journal.pone.0124195. eCollection 2015.© 2015 Wang et al.

**
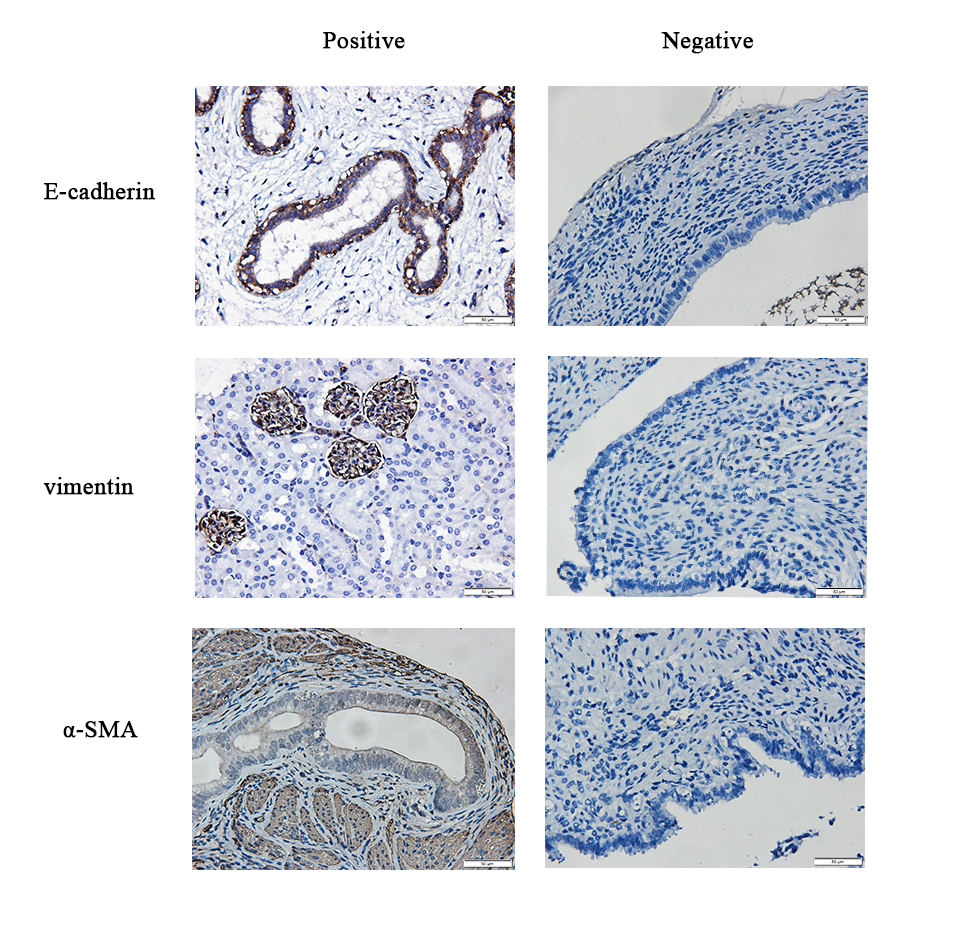
Figure S3.** Positive and negative controls for immunostaining of E-cadherin, vimentin, and α-SMA. For positive controls, human breast cancer tissues for E-cadherin, mouse kidney tissues were used for vimentin, and mouse adenomyotic tissues for α-SMA. For negative controls, endometriotic lesions were used. Vimentin and α-SMA showed positive staining in the cytoplasm, while E-cadherin was stained positive in the cell membrane. The controls all showed negative staining. Magnification: 400×; Scale bar: 50 μm.
